# Depression, anxiety and stress in taxi drivers: a systematic review of the literature

**DOI:** 10.1007/s00420-024-02117-4

**Published:** 2025-01-24

**Authors:** Marta Marín-Berges, Enrique Villa-Berges, Pablo A. Lizana, Alejandro Gómez-Bruton, Isabel Iguacel

**Affiliations:** 1 IBiOPS, Instituto de Investigación Sanitaria Aragón, Universidad de Zaragoza, Zaragoza, Spain; 2 Laboratory of Epidemiology and Morphological Sciences, Instituto de Biología, Pontificia Universidad Católica de Valparaíso, Valparaíso, 2373223 Chile; 3 EXER-GENUD (Growth, Exercise, Nutrition and Development), Faculty of Health and Sport Sciences, University of Zaragoza, Zaragoza, Spain; 4 Centro de Investigación Biomédica en Red de Fisiopatología de la Obesidad y Nutrición (CIBEROBN), Instituto de Salud Carlos III, Madrid, Spain; 5 Instituto Agroalimentario de Aragón (IA2), Zaragoza, Spain; 6 NUTRI-GENUD (Growth, Exercise, Nutrition and Development), Faculty of Health Sciences, Zaragoza, University of Zaragoza, Zaragoza, 50009 Spain

**Keywords:** Stress, Anxiety, Depression, Occupational stress, Psychological distress

## Abstract

**Purpose:**

Mental health is a global public health challenge, with mental disorders being a major cause of morbidity. Particularly, taxi drivers face unique challenges related to long working hours, economic instability, and hazardous working conditions. To summarise the existing scientific literature on mental disorders in taxi drivers and identify associated variables.

**Methods:**

PubMed, Scopus and Web of Science databases were examined from inception to April 2024 following the PRISMA guidelines. Two authors independently selected original studies. We included observational studies published in English or Spanish or Portuguese, which assessed the mental health of taxi drivers. The Quality Assessment Tool for Observational Cohort and Cross-Sectional Studies of the National Heart, Lung, and Blood Institute (NHBLI) was used to assess the quality of the articles.

**Results:**

From an initial pool of 618 studies, eleven met the inclusion criteria and were included in the present systematic review. The findings indicate a considerable prevalence of mental health issues among taxi drivers in comparison to the general population. The prevalence of depression ranged from 14.3 to 60.5% and were driven by a number of factors, including perceived mental strain, lack of respect from operators, a stressful personal life, insufficient sleep, poor working conditions, work-family conflict and low work engagement. Anxiety was reported by 24.1–47% of drivers, with a lack of sufficient sleep being identified as a primary contributing factor. The prevalence of stress ranged from 19 to 55%, with key contributing factors including discrimination, smoking, limited language proficiency, sleep disorders and younger age. Furthermore, 33% of drivers displayed elevated levels of psychological distress, frequently linked to traumatic experiences and occupational hazards.

**Conclusions:**

Rates of depression, anxiety, stress and psychological distress are higher in taxi drivers than in general population, therefore prevention strategies should target this group.

**Systematic Review Registration:**

PROSPERO registration no. CRD42023360073.

**Supplementary Information:**

The online version contains supplementary material available at 10.1007/s00420-024-02117-4.

## Introduction

Mental health is a public health problem (Li et al. 2023; Reijneveld 2005) with mental disorders representing the leading cause of non-fatal morbidity worldwide (Whiteford et al. 2013). According to the World Health Organization (WHO) (World Health Organization 2022a), in 2019, one in eight people in the world (equivalent to 970 million people) had a mental disorder. The most common mental disorders are anxiety and depression, which increased from 25 to 27% in the first year of the pandemic caused by the COVID-19 (Santomauro et al. 2021).

Allande-Cussó et al. (2022) identified four risk factors that can affect the mental health of professionals, such as individual factors (coping capacity, management of emotions and thoughts, or personality type), socio-cultural and organizational factors (work overload, working conditions, health and mental health policies of each country, health assets), biological factors (inherited genetics, nutritional deficiencies or exposure) and environmental factors (pollutants).

This highlights the need to understand how specific occupations, crucial to the functioning of society yet sometimes neglected, impact the mental health of those who work in them. Each occupation has its own unique characteristics, and therefore the factors involved will differ. For professional drivers, lorry drivers, taxi drivers, motorbike taxi drivers, bus or tram drivers, in addition to the aforementioned factors, there is the added pressure of performing their job efficiently under sometimes adverse due to the weather, road traffic, working hours or mileage (Arias-Meléndez et al. 2022).

In the specific case of taxi drivers, in addition to the logistical aspects of their work, they face unique challenges such as: operating in urban areas with more intersections, roundabouts, and interactions that require higher mental workload and quick problem-solving (Verwey 2000; Zeitlin 1995); working in a solitary job with continuous social interaction with passengers; long working hours (Wang et al. 2019), leading to less sleep (Firestone and Gander 2010), drowsiness and fatigue (Meng et al. 2015); economic instability (Del Nido 2020) related to meeting financial goals and covering taxi-related expenses (maintenance, insurance, or licenses); hazardous working conditions including driving accidents (Hitosugi et al. 2015; Jaydarifard et al. 2023), violence (Gilbert 2011) and high mortality rates (Moracco et al. 2000); taxi-associated health conditions such as sedentary lifestyles (McNeill et al. 2019), cardiovascular problems (Brucker et al. 2014; Elshatarat and Burgel, 2016), respiratory problems (Hachem et al. 2019), increased risk of cancer (Di Cesare et al. 2020), infertility (Figá-Talamanca et al. 1996) or musculoskeletal disorders (Abledu et al. 2014; Kobayashi et al. 2005); and the advent of new businesses through mobile applications (Chang 2017), increasing competitiveness (Borowiak 2019) among others.

Despite the acknowledged importance of these factors, research specifically examining the mental health of taxi drivers as a distinct occupational group remains scarce. Existing studies often fail to consider the multifaceted challenges faced by this profession. This systematic review aims to address this gap by synthesizing the scientific literature on mental disorders, particularly depression, anxiety, and stress, among taxi drivers. Additionally, it seeks to identify associated variables, thereby advancing the understanding of mental health within this occupational group and providing valuable insights for researchers, healthcare professionals, and policymakers.

## Materials and methods

The present systematic review was registered in the PROSPERO database (ID number: CRD42023360073) and followed the systematic review methodology proposed in the PRISMA (Preferred Reporting Items for Systematic reviews and Meta-Analyses) statement (Online Resource 1) (Page et al. 2021). A specific question was constructed according to the PECOS (Participants, Exposure, Control, Outcomes, Study Design) principle (Table 1).

**Table 1:** PECOS criteria for inclusion and exclusion of studies

Participants	Taxi drivers > 18 years old and < 70 years old
Exposure	Driving a taxi during at least 20 h/week
Control/comparator group	No taxi driver/No control group
Outcomes	Mental health (depression, anxiety, stress, mental disorders)
Study design	Cohort studies, cross-sectional studies, case-control studies, randomized controlled trials, and nonrandomized controlled trials

A systematic search of the literature was carried out using the PubMed, Scopus and Web of Science (from database inception to April 2024. When possible, the search included a vocabulary thesaurus (list of MeSH terms in PubMed). First, the taxi terms were combined as follows: “Taxi driver” OR “Cab driver” OR “Taxicab” OR “Taxi”. Secondly, mental outcome terms were combined as follows: “Depressi*” OR “Mental disorde*” OR “Mental health” OR “anxy*” OR “Stress*”. Finally, both taxi and mental outcome terms were combined with ‘AND’. The filters “articles,” and “in English or Spanish,” were applied when possible. There was no restriction based on date of publication.

The detailed search for each database is presented in Online Resource 2. Grey literature’ was not included. Bibliographies of included articles were searched to identify additional studies that may have met the search criteria but were not found in the search results. No further articles were found that met the inclusion-exclusion criteria. The data from the 3 databases were downloaded in Excel format and merged into a single file so that duplicates could be removed and the exclusion process could continue. Two authors (M. M and E. V) independently searched each database to obtain publications. Agreement between the authors was found for 90% of the publications, while remaining discrepancies were resolved by discussion. Relevant articles were obtained in full and assessed against the inclusion and exclusion criteria.

### Inclusion criteria

The following inclusion criteria were applied: (1) original studies; (2) studies performed in humans; (3) studies written in English or Spanish; (4) studies that include participants between 18 and 70 years of age The age limit of 70 was included in the criteria because individuals over the age of 70 are typically retired. The inclusion of older taxi drivers could potentially introduce bias into the results, as the findings might be influenced by age-related factors or the economic necessity to maintain employment, rather than being solely attributable to the taxi driver role itself.

### Exclusion criteria

The following exclusion criteria were applied: (1) articles that did not provide original data (e.g., systematic reviews, meta-analyses, literature reviews); (2) case reports; (3) other professional drivers (e.g., motorcycle taxi, truck, bus, rickshaw) and (4) articles that did not include a validated scale/questionnaire.

### Data extraction

After reviewing all relevant literature, depression, anxiety, stress and psychological distress were identified as a mental health outcome. For each study that included a mental health outcome, relevant data were extracted (Table 2) (Burgel and Elshatarat 2019; Davidson et al. 2018, 2020; Djindjic et al. 2013; Jovanović et al. 2008a; Lui et al. 2023; Maguire et al. 2006; Mirpuri et al. 2020, 2021; Rathi et al. 2019; Shin and Jeong 2021), including the number of participants, sex, mean age, instruments used to assess outcomes, study design and quality assessment score. The instruments used to assess outcomes were either professionally administered or self-reported by study participants. The following tools were used to assess depression: the Depression, Anxiety and Stress-Depression Scales (DASS-D), the Centre for Epidemiological Studies Depression Scale and the World Health Organization Well-Being Index (WHO-5). The Depression, Anxiety, Stress-Anxiety, Stress-Anxiety Scale (DASS-A) and the State-Trait Anxiety Inventory (STAI) were used to assess anxiety. To assess stress, the Stress, Anxiety and Depression-Stress Scale (DASS-S), Perceived Stress Scale (PSS-10) and Occupational Stress Index (OSI) were used. The Kessler Psychological Distress Scale (K-10) was used to assess psychological distress.

Other information extracted included the following: (1) number of immigrants, (2) mileage, (3) hours of work, (4) days per week of work, (5) predominant work shift, (6) average years of work, (7) hours of sleep, (8) compatibility with other jobs, (9) predominant marital status, (10) taxi ownership, (11) education, (12) tobacco use, (13) alcohol use, (14) physical activity, (15) obesity, and (16) possession of health insurance. This information is detailed in Table 2.

Assessment of methodological quality.

The Quality Assessment Tool for Observational Cohort and Cross-Sectional Studies of National Heart, Lung, and Blood Institute (NHBLI) (*Study Quality Assessment Tools | NHLBI*,* NIH*, n.d.) as used to assess the quality of the articles.

This tool evaluates 14 critical aspects, including the clarity of objectives, population selection, sample adequacy, and the measurement of key variables. It also assesses the appropriateness of statistical methods and the consideration of potential confounding factors. Each aspect is rated using straightforward categories: ‘yes’, ‘no’, ‘not applicable’, or ‘not reported’.

The selection of this tool is justified by its specificity to the type of articles being reviewed, its clear structure, and its systematic approach to identifying strengths and weaknesses in each analyzed study. Moreover, its application ensures that the conclusions drawn from this review are grounded in robust evidence, which is essential for generating reliable and actionable recommendations (*Background: Development and Use of Study Quality Assessment Tools | NHLBI*,* NIH*, n.d.).

The quality of the assessment has 1 study equal to 57.4%, 1 study 50%, 7 studies 42.8% and the remaining 2 studies 35.7% as shown in Online Resource 3.

## Results

### Search summary

**Fig. 1:**
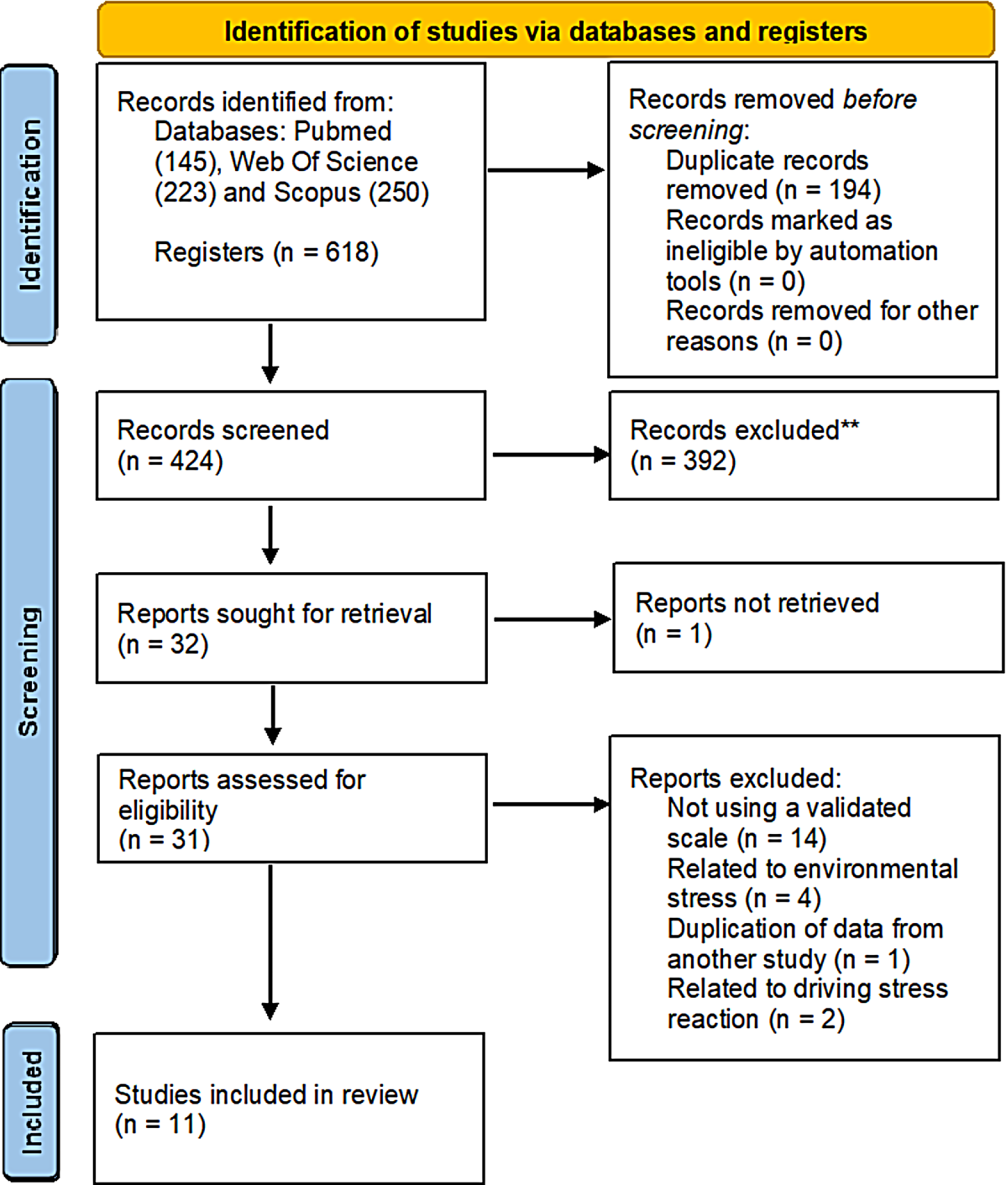
PRISMA 2020 flow diagram

A total of 618 articles were extracted from PubMed (145), Scopus (250) and Web Of Science (223). From these, 194 manuscripts were duplicates, 392 manuscripts were excluded after reading the title and abstract as they did not meet the inclusion and exclusion criteria. Two manuscripts were not accessed. 31 full-text articles were assessed for eligibility, of which 21 were excluded (Fig. 1): 14 did not include a validated scale (Ba et al. 2018; Bawa and Srivastav 2013; Berrones-Sanz and Araiza-Diaz 2019; Bulduk et al. 2014; Chen et al. 2005; Dai et al. 2023; Kumari and Sidhu 2016; Murray et al. 2019; Peng et al. 2022; Shahrukh et al. 2020; Subedi and Rosenberg 2016; Tokars et al. 2012; Wang and Delp 2014; Yang et al. 2014), four assessed environmental stress but not individual stress (Chen and Sih 2024; Tàpia-Caballero et al. 2022; Useche et al., 2018), one duplicated data from another already included (Burgel and Elshatarat 2017) and two driving stress reaction (Montoro et al. 2018; Öz et al. 2010). Consequently, a total of 11 studies were included in the present systematic review.

### Search strategy and study selection

After applying the exclusion criteria, 11 articles were included in this review: four examined depression (Burgel and Elshatarat 2019; Davidson et al. 2020; Rathi et al. 2019; Shin and Jeong 2021), three anxiety (Davidson et al. 2020; Maguire et al. 2006; Rathi et al. 2019), eight stress (Davidson et al. 2020; Djindjic et al. 2013; Jovanović et al. 2008a; Lui et al. 2023; Maguire et al. 2006; Mirpuri et al. 2020, 2021; Rathi et al. 2019) with two of them examining occupational stress (Djindjic et al. 2013; Jovanović et al. 2008a) and one the level of psychological distress (Davidson et al. 2018). Sample sizes ranged from 18 to 1105 people per study, with the present review including a total of 2,892 participants (1.03% female). The country of origin of the studies included were Australia (Davidson et al. 2018, 2020), USA (Lui et al. 2023; Mirpuri et al. 2020, 2021), India (Rathi et al. 2019), UK (Maguire et al. 2006), Serbia (Djindjic et al. 2013; Jovanović et al. 2008a) y Korea (Shin and Jeong 2021). All the included studies were cross-sectional (Burgel and Elshatarat 2019; Davidson et al. 2018, 2020; Djindjic et al. 2013; Jovanović et al. 2008a; Lui et al. 2023; Maguire et al. 2006; Mirpuri et al. 2020, 2021; Rathi et al. 2019; Shin and Jeong 2021).

**Table 2:** Characteristics of included studies

Reference and country	Participants		Outcomes of mental health				Study design	Other information	QA
	No. of taxi drivers (mean age ± SD)	Sex (male/fem.)	Outcome	Instrument used	Mean ± SD	Variables related to mental health			
Burgel et al. (2019) United State	130 (45.3 ± 10.75)	M 122/F 8	Depression	CES-D	Overall sample: 14.6 ± 8.7 (high risk of depression) 38% Depression	Mental perception of effort: *r* = 0.389, *P* < 0.001 No respect by dispatcher (OR = 4.729; *p* = 0.012) Personal stressful life events (continuous score (0-6), higher scores = higher stress) (OR = 2.216; *p* = 0.006)	Cross-Sectional	IMM: 55% - KM/MI: 539.26 ± 200.9 mi/week - WH: 41(± 13) h/w - DW: NR - SHIFT: nightly 51% - WY: 9,7 ± 8.4 - SLEEP: <6 h 36% - OW: 19% - MS: married/partnered 54% - PROPERTY: NR - EDUCATION: non-graduates 61.5% - TABACCO: 36% - ALCOHOL: infrequent 26% - PA: no 33% - OBESITY: 25% - HI: 42%	50%
Davidson et al. (2018) Australia	380 (40.7 ± 12.12)	M 358/F 22	Depression Anxiety	K10	Overall sample: 24.73 ± 8.04 (likely to have a mild disorder) 33.3% = high levels 28.3% = very high levels 14.6%= low range.	- Seen someone killed/badly injured (OR = 1.84; *p* = 0.014). - Life-threatening car accident (OR = 2.11; *p* = 0.012). - Other life-threatening accident (OR = 5.13; p = < 0.001). - Mugged/threatened (OR = 2.44; *p* = 0.002). - Beaten up badly (OR = 3.09; *p* = 0.002). - Any other trauma (OR = 2.26; *p* = 0.008). - Experienced three or more PTEs (OR = 5.24; p = < 0.001). - Visited a specialist (OR = 1.85; *p* = 0.017).	Cross-Sectional	-IMM: 92.8% - KM/MI: NR - WH: 51.6(± 19.6) h/w - DW: NR - SHIFT: nightly 77% - WY: 10,7 ± 8.67 - SLEEP: NR - OW: 22.4% - MS married/partnered 74.4% - PROPERTY NR - EDUCATION: Bachelor’s degree or higher 45,7% - TABACCO: NR - ALCOHOL: NR - PA: NR - OBESITY: NR - HI: NR	35.7%
Davidson et al. (2020) Australia	46 (32.2 ± 9.8)	M 46	Depression Anxiety Stress	DASS-D DASS-A DASS-S	Overall sample: Depression 6.15 ± 8.96 (mild depression) - normal 75.7%. - mild 13.5%. - moderate 5.4%. -Severe 0. - extremely severe 5.4%. Anxiety 5,75 ± 6,08 (moderate anxiety) - normal 81%. - mild 4.8%. - moderate 7.1%. - severe 7.3%. - extremely severe 4.9%. Stress 6.88 ± 6.71 (normal stress) - normal 81%. - mild 4.8% moderate 7.1%. - moderate 7.1%. - severe 0; - extremely severe 7.1%	Pre-validation data collection. Variables related to mental health: NR		- IMM: 95% - KM/MI: NR - WH: NR - DW: NR - SHIFT: NR - WY: NR - SLEEP: NR - OW: NR - MS: married 74% - PROPERTY: NR - EDUCATION: Diploma certificate 43% - TABACCO: NR - ALCOHOL: NR - PA: NR - OBESITY: NR - HI: NR	42.8%
Djindji et al. (2013) Serbia	122 (42.98 ± 10.5)	M 122	Occupational stress Index	OSI	Overall sample: Total: 71.61 ± 4.4 (mild stress) High demands: 17.89 ± 2.7 Strictness: 16.45 ± 1.6 Conflict: 15.71 ± 1.6 Underload: 3.11 ± 0.8 Avoidance: 7.83 ± 1.5 Extrinsic time pressure: 6.55 ± 1.1 Noxious exposures: 4.47 ± 0.5	Are global data, there is no separation by driver type Variables related to mental health: NR	Cross-Sectional	- IMM: NR - KM/MI: NR - WH: NR - DW: NR - SHIFT: NR - WY: 18.64 ± 10.0 - SLEEP: NR - OW: NR - MS: NR - PROPERTY: NR - EDUCATION: NR - TABACCO: 52.4% - ALCOHOL: NR - PA: NR - OBESITY: NR - HI: NR	57.4%
Jovanović et al. (2008a, b) Serbia	34	M 34	Occupational stress Index	OSI	Overall sample: Total: 61.5 ± 9.8 (mild stress) High demands: 15.3 ± 2.1 Strictness: 12.3 ± 2.8 Conflict: 1.5 ± 38.5 Underload: 1.7 ± 1.1 Avoidance: 7.8 ± 0.3 Extrinsic time pressure: 7.7 ± 1.1 Noxious exposures: 6.2 ± 3.7	Are global data, there is no separation by driver type Variables related to mental health: NR	Cross-Sectional	- IMM: NR - KM/MI: NR - WH: NR - DW: NR - SHIFT: NR - WY: NR - SLEEP: NR - OW: NR - MS: NR - PROPERTY: NR - EDUCATION: NR - TABACCO: NR - ALCOHOL: NR - PA: NR - OBESITY: NR - HI: NR	42.8%
Lui et al. (2023) United State	1105 (45.47 ± 12.00)	M 1105	Stress	PSS-10	Overall sample: 45.6% Moderate/high stress 52.9% Low stress 1.5% Missing	Variables related to mental health: NR	Cross-Sectional	- IMM: 91.9% - KM/MI: NR - WH: NR - DW: NR - SHIFT: daytime 42.6% - WY: NR - SLEEP: NR - OW: NR - MS: pareja 61% - PROPERTY: NR - EDUCATION: universitarios 30.4% - TABACCO: 14% - ALCOHOL: 38.5% - PA: NR - OBESITY: NR - HI: 49.90%	42.8%
Maguire et al. (2006) UK	18 (39.33 ± 4.49)	M 18	Stress Anxiety	PSS-10 STAI-S STAI-T	Overall sample: Perceived stress sale 16.39 ± 7.96 (moderate stress) STAI-S 29.50 ± 7.99 (no/low anxiety) STAI-T 38.11 ± 12.31 (moderate anxiety tendency)	Variables related to mental health: NR	Cross-Sectional	- IMM: NR - KM/MI: NR - WH: NR - DW: NR - SHIFT: NR - WY: 10.94 ± 5.25 - SLEEP: NR - OW: NR - MS: married 74% - PROPERTY: NR - EDUCATION: school dropouts 16.50 ± 0.90 - TABACCO: NR - ALCOHOL: NR - PA: NR - OBESITY: NR - HI: NR	42.8%
Mirpuri et al. (2020) United State	535 (44.09 ± 11.33)	M 535	Stress	PSS-10	Overall sample: 12.5 ± 7.0 (low stress)	Everyday discrimination (OR = 2.45, p = < 0.0001) Age: older age less stress: (*r* = -0.18, *p* < 0.01)	Cross-sectional	- IMM: 96% - KM/MI: NR - WH: NR - DW: NR - SHIFT: NR - WY: NR - SLEEP: NR - OW: NR - MS: NR - PROPERTY: NR - EDUCATION: 12 grade 31.1% - TABACCO: NR - ALCOHOL: NR - PA: NR - OBESITY: NR - HI: 74%	42.8%
Mirpuri et al. (2021) United State	252 (44.92 ± 11.21)	M 252	Stress	PSS-10	Overall sample: 13.9 ± 6.87 (low stress) 46% =low stress 52% =moderate stress 3% =high stress	English proficiency (β= − 0.222; *p* = 0.001) Sleep disturbances (β = 525; *p* = 0.000) Smoking (β = 901; *p* = 0.013)	Cross-sectional	- IMM: 98% - KM/MI: NR - WH: NR - DW: NR - SHIFT: daytime 6% - WY: NR - SLEEP: 6.8 ± 1.18 - OW: NR - MS: married 82% - PROPERTY: 38.1% - EDUCATION: secondary 44% - TABACCO: 17.9% - ALCOHOL: NR - PA: inactivity 40% - OBESITY: NR - HI: NR	42.8%
Rathi et al. (2019) India	134 (32.51 ± 7.92)	M 134	Depression Anxiety Stress	DASS-D DASS-A DASS-S	Overall sample: 60.5% depression 47% anxiety 36.5% stress	Stress: Lack of sleep (*p* = 0.009) Irritation (*p* = 0.004) Stress, anxiety, depression: sleep duration < 8 h (*p* = 0.02)	Cross-Sectional	- IMM: 64.18% - KM/MI: 2225 ± 54.91 km/day - WH: 12 ± 2.64 day - DW: 6 días 59.70% - SHIFT: NR - WY: ≥ 1 year 57.46% - SLEEP: 5–7 h 67.2% - OW: NR - MS: married 83.58% - PROPERTY: car 70.15% - EDUCATION: secondary 33.58% - TABACCO: 32.58% - ALCOHOL: 1x/week 31.34% - PA: inactivity 83.58% - OBESITY: NR - HI: NR	42,8%
Shin et al.(2021) Corea	54,9	M 136	Depression	WHO-5	Overall sample: 40.4% depression	Poor work situation (*r* = 0.250; p = < 0.001). Conflict between work and family (*r* = 0.117; *p* = 0.019). Depression has a significant effect on poor work engagement (*r* = 0.524; p = < 0.001).	Cross-sectional	- IMM: NR - KM/MI: NR - WH: NR - DW: NR - SHIFT: NR - WY: NR - SLEEP: NR - OW: NR - MS: NR - PROPERTY: NR - EDUCATION: NR - TABACCO: NR - ALCOHOL: NR - PA: NR - OBESITY: NR - HI: NR	35.7%

Abbreviations Instrument used: CES-D, Center for Epidemiologic Studies Depression Scale; DASS-A, Depression Anxiety Stress Scale–Anxiety; DASS-D, Depression Anxiety Stress Scale–Depression; DASS-S, Depression Anxiety Stress; K10, Kessler Psychological Distress Scale; OSI, Occupational Stress Index; PS-10, Perceived Stress Scale-10; STAI, State-Trait Anxiety Inventory; STAI-S, State trait anxiety inventory state; STAI-T, State trait anxiety inventory trait; WHO-5, 5-item World Health Organization Well Being Index

Abbreviations Other information: DW, Days Week; HI, Health Insurance; IMM, Immigrants; KM/MI, Kilometres/milles; MS, Marriage Situation; NR, Non-Report; OW, Other Works; PA, Physical Activity WH, Work Hours; WY, Work Years

Cut-off values of the scale: CES-D, High risk of depression ≥ 16 de 60; DASS-D, 0–4: normal, 5–6: mild, 7–10: moderate, 11–13: severe, ≥ 14: extremely severe; DASS-A, 0–3: normal, 4: mild, 5–7: moderate, 8–9: severe, ≥ 10: extremely severe; DASS-S, 0–7: normal, 8–9: mild, 10–12: moderate, 13–16: severe, ≥ 17: extremely severe; K10, 10–19: likely to be well, 20–24: likely to have a mild disorder, 25–29: likely to have a moderate disorder, 30–50: likely to have a severe disorder; OSI, ≥ 76: mild stress, 77–152: moderate stress, 153–230: severe stress; PS-10, 0–13: low stress, 14–26: moderate stress, 27–40: high perceived stress; STAI-S, 20–37: no/low anxiety, 38–44: moderate anxiety, 45–80: high anxiety; STAI-T, 20–37: tendency no/low anxiety, 38–44: trend moderate anxiety, 45–80: tendency high anxiety; WHO-5, ≥ 13: possible depression

### Taxi driver characteristics

#### Socio-demographic factors

As shown in Table 2, nine studies included only men (Davidson et al. 2020; Djindjic et al. 2013; Jovanović et al. 2008a; Lui et al. 2023; Maguire et al. 2006; Mirpuri et al. 2020, 2021; Rathi et al. 2019; Shin and Jeong 2021) in the other two, female accounted for 1.9% (Davidson et al. 2018) and 6.15% (Burgel and Elshatarat 2019). Six of the eleven studies reported mean ages over 40 years (Davidson et al. 2018; Djindjic et al. 2013; Lui et al. 2023; Mirpuri et al. 2020, 2021; Shin and Jeong 2021). The percentage of taxi drivers who were immigrants ranged from 55% (Burgel and Elshatarat 2019), 64.8%(Rathi et al. 2019) to over 90% in most studies (Davidson et al. 2018, 2020; Lui et al. 2023; Mirpuri et al. 2020, 2021). In the studies that assessed marital status (Burgel and Elshatarat 2019; Davidson et al. 2018; Lui et al. 2023; Maguire et al. 2006; Mirpuri et al., 2021; Rathi et al. 2019), most participants reported being married or in a relationship (Burgel and Elshatarat 2019; Davidson et al. 2018; Lui et al. 2023; Maguire et al. 2006; Mirpuri et al., 2021; Rathi et al. 2019) with percentages ranging from 55% (Burgel and Elshatarat 2019) to 83.38% (Rathi et al. 2019).

#### Occupational factors

The average number of hours worked was analyzed in 3 studies. Of these, two reported hours per week: 41 h (Burgel and Elshatarat 2019) and 51.6 h per week (Davidson et al. 2018). The third study reported 12 h per day (Rathi et al. 2019). Additionally, only two studies assessed compatibility with another job, with affirmative responses of 22.4% (Davidson et al. 2018) and 19% (Burgel and Elshatarat 2019).

#### Level of depression, anxiety, stress and occupational distress

Subdivided results are presented below for each of the dependent variables: depression, anxiety, stress and occupational distress. Table 3 schematically presents the mean score of these variables along with the reported prevalence.

#### Level of depression

Four studies examined the level of depression in taxi drivers (Burgel and Elshatarat 2019; Davidson et al. 2020; Rathi et al. 2019; Shin and Jeong 2021). Of these four studies, two used the Depression, Anxiety and Stress Scale (DASS-21), depression subscale (Davidson et al. 2020; Rathi et al. 2019), one used the Centre for Epidemiological Studies Depression Scale (CES-D) (Burgel and Elshatarat 2019) and one study used World Health Organization Well-Being Index (WHO-5) (Shin and Jeong 2021). The percentage of taxi drivers with symptoms of depression ranged from 14.3% (Davidson et al. 2020), 38% (Burgel and Elshatarat 2019), 40.4% (Shin and Jeong 2021) to 60.5% (Rathi et al. 2019). Additionally, depression was classified as high 14.6 ± 8.7 (Burgel and Elshatarat 2019) in one study and normal 6,15 ± 8,96 (Davidson et al. 2020) in another study. Factors associated with depression included perceived mental strain and lack of respect from dispatchers (Burgel and Elshatarat 2019), personal stressful life (Burgel and Elshatarat 2019), sleep duration less than 8 h (Rathi et al. 2019), poor work situation, work-family conflict and low work engagement (Shin and Jeong 2021). One study focused on the validation of the Driving to Health site and did not assess anxiety associations (Davidson et al. 2020).

#### Anxiety level

Three studies examined anxiety levels among taxi drivers (Davidson et al. 2020; Maguire et al. 2006; Rathi et al. 2019). Two studies used the Depression, Anxiety and Stress Scale (DASS-21) anxiety subscale (Davidson et al. 2020; Rathi et al. 2019), while one used The Perceived Stress Scale State-Trait Anxiety Inventory: State trait anxiety inventory state and State trait anxiety inventory trait subscales (Maguire et al. 2006). Anxiety prevalence ranged from 24.1%(Davidson et al. 2020) to 47%(Rathi et al. 2019) of the taxi drivers had some degree of anxiety. Anxiety levels were categorized as null/low 29.50 ± 7.99(Maguire et al. 2006) or moderate 5.75 ± 6.08 (Davidson et al. 2020). Only one study examined potential factors contributing to anxiety, identifying sleep duration of less than 8 h as a significant factor (Rathi et al. 2019).

#### Stress level

Eight studies examined the stress level of taxi drivers (Davidson et al. 2020; Djindjić et al., 2013; Jovanović et al. 2008; Lui et al. 2023; Maguire et al. 2006; Mirpuri et al. 2020, 2021; Rathi et al. 2019). Of these, four used the Perceived Stress Scale (PSS-10) (Lui et al. 2023; Maguire et al. 2006; Mirpuri et al. 2020, 2021), two used the Depression, Anxiety and Stress Scale (DASS-21) stress subscale (Davidson et al. 2020; Rathi et al. 2019) y two Occupational Stress (OSI) (Djindjic et al. 2013; Jovanović et al. 2008a). The prevalence of stress in taxi drivers was 19% (Davidson et al. 2020), 36.5% (Rathi et al. 2019), 45.6% (Lui et al. 2023) y 55% (Mirpuri et al. 2021). And their stress levels were low 12.5 ± 7.0 (Mirpuri et al. 2020), 13.9 ± 6.87 (Mirpuri et al. 2021), normal 6.88 ± 6.71 (Davidson et al. 2020) or moderate 16.39 ± 7.96 (Maguire et al. 2006).

In the specific case of occupational stress, both studies showed that taxi drivers had a level of mild stress 71.61 ± 4.4 (Djindjic et al. 2013) y 61.5 ± 9.8 (Jovanović et al. 2008).

Three studies assessed factors that could contribute to stress (Mirpuri et al. 2020, 2021; Rathi et al. 2019) such as daily discrimination (Mirpuri et al. 2020); younger age(Mirpuri et al. 2020), language proficiency in the country of work (Mirpuri et al. 2021), smoking (Mirpuri et al. 2021), sleep disturbance (Mirpuri et al. 2021), lack of sleep (Rathi et al. 2019) o sleep duration of less than 8 h (Rathi et al. 2019), as well as irritation (Rathi et al. 2019). Five studies did not investigate possible stressors (Davidson et al. 2020; Djindjic et al. 2013; Jovanović et al. 2008a; Lui et al. 2023; Maguire et al. 2006).

#### Level of psychological distress

One study analysed the level of psychological distress (Davidson et al. 2018) using the Kessler Psychological Distress Scale (K10). The mean score was 24.73 ± 8.04, indicating a moderate level of psychological distress. Factors associated with psychological distress included: witnessing someone’s death or severe injury, involvement in life-threatening traffic accidents or other serious accidents, experiencing assault, threats, or abuse, exposure to other traumatic events, experiencing three or more potentially traumatic events, and consulting with a specialist.

**Table 3:** Results subdivided for level of depression, anxiety, stress and occupational distress

	Mean ± SD	%
Depression		
Burgel et al. (2019) CES-D	14.6 ± 8.7 = High risk	38% = Depression
Davidson et al. (2020) DASS-D	6.15 ± 8.96 = Normal	14.3% = Depression
Rathi et al. (2019) DASS-D	NR	60.5% = Depression
Shin et al. (2021) WHO-5	NR	40.4% = Depression
*Anxiety*		
Davidson et al.(2020) DASS-A	5.75 ± 6.08 = Moderate	24,1% = Anxiety
Maguire et al. (2006) STATE	State: 29.59 ± 7.99 = No/low Anxiety Trait: 38.11 ± 12.31 = Moderate tendency to Anxiety	NR
Rathi et al.(2019) DASS-A	NR	47% = Anxiety
*Stress*		
Davidson et al.(2020) DASS-S	6.88 ± 67.1 = Normal	19% = Stress
Djindji et al.(2013)OSI	71.61 ± 4.4 = Mild	NR
Jovanović et al. (2008a, b)OSI	61.5 ± 9.8 = Mild	NR
Lui et al.(2023) PSS-10	NR	45.6% = Moderate/high Stress 52.9% = Low Stress
Maguire et al.(2006) PSS-10	16.39 ± 7.96 = Moderate	NR
Mirpuri et al. (2020) PSS-10	12.5 ± 7.0 = Low	NR
Mirpuri et al. (2021) PSS-10	13.9 ± 6.87 = Low	46% = low Stress 52% = moderate Stress 3% = high Stress
Rathi et al.(2019) DASS-S	NR	36.5% = Stress
*Psychological distress*		
Davidson et al.(2018) K10	24.73 ± 8.04 = Likely to have a mild disorder	33.3% = High levels Psychological distress 28.3% = Very high levels Psychological distress 14.6% = low range Psychological distress

## Discussion

The aim of this review was to systematically summarize the literature on the mental health of taxi drivers. The results of this systematic review indicate that taxi drivers carry a significant burden of mental health problems, including elevated levels of depression, anxiety, stress and psychological distress compared with the general population (World Health Organization 2022). It is crucial to identify and address associated risk factors, both occupational and personal, to enhance the mental health and well-being of taxi drivers. Anxiety, depression, and stress are closely intertwined and frequently co-occur within individuals (Lovibond 1995). Furthermore, chronic stress can precipitate symptoms of both anxiety and depression, establishing a cycle where each condition exacerbates the other (Hammen 2005).

The prevalence of depression among taxi drivers significantly exceeds that of the general population. For instance, in India, the prevalence of depression was 60.5% among taxi drivers (Rathi et al. 2019), compared to 4% in the general Indian population (Gururaj et al. 2016). In the United States, the rate of depression among taxi drivers was 38% (Burgel and Elshatarat 2019), contrasting with 8.1% in the adult population (Brody et al. 2018). Similarly, in Australia, 24.3% of taxi drivers experienced depression (Davidson et al. 2020), which is notably higher than the 4.9% observed in the general Australian population (Australian Bureau of Statistics, n.d.). Factors associated with depression include perceived mental strain (Burgel and Elshatarat 2019), lack of respect from dispatchers (Burgel and Elshatarat 2019), stressful personal life events (Burgel and Elshatarat 2019), sleep duration less than 8 h (Rathi et al. 2019), adverse working conditions and work-family conflict (Rathi et al. 2019). These factors can significantly impact the work engagement of taxi drivers (Shin and Jeong 2021). Although the instruments used are not diagnostic tools, their content allows meaningful connections to be made with the Diagnostic and Statistical Manual of Mental Disorders, Five Edition (DSM-5)(American Psychiatric Association 2013) criteria, the most recognised diagnostic standard for mental disorders. The DASS-D includes items that assess key aspects of major depressive disorder, such as depressed mood (‘I felt sad and depressed’) and loss of interest or pleasure in activities (‘I could not get excited about anything’). For its part, the CESD explicitly incorporates DSM-5 domains associated with major depression, such as recurrent thoughts of death or suicidal ideation (‘I wished I was dead’), difficulty concentrating (‘I couldn’t concentrate’) and loss of interest in usual activities (‘I lost interest in my activities’).

Alongside depression, anxiety is also a prevalent mental disorder among taxi drivers, with considerably higher rates than in the general population. In India, 47% of taxi drivers experienced some form of anxiety (Rathi et al. 2019), compared to 2.57% in the general Indian population (Manjunatha et al. 2022). In Australia, the prevalence of anxiety among taxi drivers was 24.1% (Davidson et al. 2020), exceeding 17.2% (Australian Bureau of Statistics, n.d.) in the general Australian population. Research indicates that sleep duration, particularly when less than 8 h (Rathi et al. 2019), is a significant factor contributing to anxiety levels among taxi drivers. As in the case of depression, the DASS-A and STAI instruments show a significant relationship with the DSM-5 (American Psychiatric Association 2013) diagnostic criteria for anxiety disorders. In the DASS-A, items such as ‘I felt I was on the verge of panic’, ‘It became difficult to breathe’ and ‘I felt my heart pounding even though I had not made any physical effort’ reflect symptoms of panic disorder. Other items such as ‘It was difficult to release tension’ and ‘I felt afraid for no reason’ reflect basic features of generalised anxiety disorder (GAD). On the other hand, STAI-S items such as ‘I feel scared for no apparent reason’, ‘I feel restless’ and ‘I feel tense or nervous’ match the intense emotional and physiological symptoms described for panic disorder and episodic anxiety. In the STAI-T, items such as ‘I am generally tense’, ‘I worry too much about unimportant things’ and ‘I often have disturbing thoughts’ reflect key aspects in the diagnosis of GAD.

The reviewed studies showed varying levels of stress among taxi drivers, mostly in the low to moderate range (Davidson et al. 2020; Djindjic et al. 2013; Jovanović et al. 2008a; Maguire et al. 2006; Mirpuri et al. 2020, 2021). Factors associated with stress include daily discrimination (Mirpuri et al. 2020), lack of English proficiency (Mirpuri et al. 2021), smoking (Mirpuri et al. 2021), sleep disturbance (Mirpuri et al. 2021), and insufficient sleep (Rathi et al. 2019). Additionally, stress levels appear to decrease with age, with older taxi drivers experiencing lower stress levels (Mirpuri et al. 2020). The DASS-S through items such as ‘I found it hard to relax’ reflect hyperarousal and chronic tension, symptoms aligned with DSM-5 (American Psychiatric Association 2013) stress-related disorders such as Acute Stress Disorder (ASD) and Post-Traumatic Stress Disorder (PTSD). Similarly, items such as ‘I overreacted in certain situations’ highlight irritability and overreactivity, while ‘I did not tolerate anything that interfered with my tasks’ reflects difficulties in managing external demands, linked to Adjustment Disorders. The PSS-10 with items such as ‘How often have you felt that difficulties accumulated beyond your control?’ addresses a sense of loss of control and overwhelming stress, central to disorders such as ASD and Adjustment Disorder. Questions such as ‘How often have you felt angry because things were out of your control?’ assess emotional hyper-reactivity and persistent tension, common in stress-related disorders.

Contrary to the general results, in Serbia, studies on occupational stress among taxi drivers suggested that this population experiences overall low levels of stress. Compared to other drivers (Jovanović et al. 2008) and other professions (Aryal and D’mello 2020; Desouky and Allam 2017; Jovanović et al. 2021), taxi drivers in Serbia exhibited significantly lower stress scores. The stress levels reported in Serbian taxi drivers contrast not only with those in other regions, but also with those of other drivers and professionals within the same cultural context. The perception and reporting of stress may differ in Serbia due to cultural factors. For instance, strong community resilience and close social ties, particularly in rural areas, may play a protective role in mitigating the perception of work-related stress. Additionally, cultural values that emphasize emotional strength and stoicism may lead to an underestimation or underreporting of stress levels among Serbian taxi drivers. Even within the same cultural context, differences in reported stress levels between Serbian taxi drivers and other drivers or professionals could be attributed to variations in work demands. Factors such as workload intensity, scheduling flexibility, or exposure to occupational hazards may differ between professions, influencing stress levels. These regional and occupational variations highlight the need for future research to examine the interplay between cultural, policy, and job-specific factors in shaping the perception and reporting of stress. This would provide a more comprehensive understanding of the observed differences. To measure occupational stress, the Occupational Stress Index (OSI) was used. The OSI is based on an additive load model that assesses stress-related work factors, especially those with an impact on the cardiovascular system (Belkic et al. 2004) which is considered a specific feature of high-risk jobs. This index integrates elements of Karasek’s Job Strain Model (Karasek 1979), Occupational stress among taxi drivers can be effectively contextualized through the Job Demand-Control (JDC) Model (Bakker and Demerouti 2007) a widely recognized framework for understanding work-related stress. The JDC Model suggests that the interaction between high job demands (e.g., workload, time constraints, and emotional strain) and low job control (e.g., limited autonomy over work tasks and decision-making) significantly influences stress outcomes. Taxi drivers are typically exposed to a high-demand, low-control work environment, characterized by extended working hours, unpredictable schedules, and constrained decision-making power, all of which contribute to heightened stress and adverse mental health outcomes, including anxiety, depression, and sleep disturbances. In this regard, (Useche et al., 2018), found that on public transport drivers, high levels of job strain (characterized by high psychological demands and low decision latitude) were associated with increased traffic accidents and sanctions, highlighting the broader implications of stress on both well-being and performance.

Moreover, the data on psychological distress among taxi drivers is concerning. An Australian study found that 33% of taxi drivers exhibited high levels of psychological distress (Davidson et al. 2020) compared to 15% of the general Australian population as reported in the National Survey of Mental Health and Wellbeing 2021 (Australian Institute of Health and Welfare, n.d.). This high level of psychological distress underscores the urgent need for targeted interventions to address risk factors and enhance the mental health of taxi drivers.

As evidenced in the review, mental health problems among taxi drivers are significantly influenced by both occupational and personal factors. For instance, poor sleep quality and short sleep durations (≤ 7 h per night) (Mirpuri et al., 2021; Rathi et al. 2019), are identified as critical risk factors for depression, anxiety, and occupational stress. These issues not only impair cognitive functioning but also increase susceptibility to occupational hazards such as traffic accidents and reduced job performance(Elshatarat and Burgel 2023). Additionally, working conditions—particularly during night shifts—expose drivers to heightened risks, such as aggressive or intoxicated passengers and violence (e.g., robbery or physical assault), which exacerbate stress, anxiety, and psychological hyper-alertness, ultimately deteriorating their mental and physical well-being(Burgel et al. 2014).

Drivers face cumulative trauma from repeated exposure to distressing events, including witnessing accidents or experiencing discrimination. Immigrant taxi drivers, in particular, encounter compounded challenges due to cultural and social barriers, limited access to support networks, and systemic discrimination (Facey 2003). These factors contribute to higher levels of psychological distress, affecting their quality of life and work performance.

Regional policy environments and cultural attitudes significantly shape mental health outcomes. For example, countries with stricter labor protections and better access to mental health services report more favorable outcomes among drivers compared to regions with lax policies and limited resources (Burns 2015). Furthermore, cultural attitudes toward mental health and masculinity play an important role. In certain cultures, stigma associated with seeking help, coupled with societal expectations of men as stoic providers, hinders the recognition and treatment of mental health problems (Courtenay 2000; Leong and Kalibatseva 2011).

Mental health challenges among taxi drivers are closely intertwined with physical health risks, particularly cardiovascular diseases. Chronic stress, anxiety, and depression elevate the risk of hypertension, cardiac arrhythmias, and coronary heart disease, creating a vicious cycle in which deteriorating mental and physical health reinforce each other (Elshatarat & Burgel, 2016).

### Strengths and limitations

To the authors’ knowledge, this study is the first systematic review to examine the mental health of taxi drivers in detail, providing comprehensive coverage of the topic by analyzing studies from diverse geographies and socioeconomic contexts. The inclusion of studies from various countries (Australia, USA, India, United Kingdom, Serbia, and Korea) provides a global perspective on the mental health of taxi drivers, allowing international comparisons and understanding contextual differences. The review not only documents the prevalence of mental health problems among taxi drivers but also identifies associated risk factors and contextual variables, providing valuable information for future research and intervention policies.

Despite its comprehensive scope, this study has several limitations that must be acknowledged. A first limitation is the heterogeneity of the studies in terms of methodology, assessment instruments, and sample characteristics, which makes the comparison of results and generalization difficult. For example, the use of different assessment instruments may lead to discrepancies in the prevalence and severity of reported mental health problems. In this systematic review, the included articles do not provide specific subtypes or categories of disorders such as anxiety, depression and stress, as defined in the DSM-5, nor do they distinguish between conditions such as generalised anxiety disorder, panic disorder or major depressive disorder. Instead, the studies employ a variety of diagnostic tools, as discussed in the manuscript, rather than specialist diagnoses according to DSM-5 criteria. Also, variations in sample sizes and demographic compositions may introduce biases, such as over- or under-representation of certain subgroups. Second, another important source of heterogeneity is the variation in the theoretical frameworks employed by the studies The presence or absence of theoretical models in the reviewed studies has been examined to better contextualize the findings and to highlight the limitations when comparing or generalizing the results. Some studies employ well-established frameworks, such as the Occupational Stress Index (OSI), which is grounded in models like the Job Strain (Karasek 1979) and Effort-Reward Imbalance models (Siegrist et al. 1991), enabling the exploration of links between job stress and physiological outcomes (e.g., metabolic and cardiovascular disturbances). Other studies apply frameworks such as the Self-Medication Hypothesis (Bolton et al. 2006), which explains substance use as a coping mechanism for untreated emotional issues, or the ABC Theory of Emotions (Ellis 1963), which analyzes how individual beliefs influence maladaptive behaviors. However, a notable proportion of the reviewed articles do not adopt an explicit theoretical framework. This absence limits the interpretation of causal relationships and often results in studies with a predominantly descriptive focus. Also, differences in theoretical frameworks, measurement tools, sample sizes, and sample compositions introduce significant heterogeneity, complicating direct comparisons between studies. For instance, the use of varying diagnostic tools or survey instruments can yield inconsistent results, while differences in sample size and population demographics may skew prevalence rates or associations with mental health outcomes. These methodological and theoretical discrepancies underscore the need for caution when synthesizing findings and interpreting results across studies. Furthermore, they highlight the importance of standardizing research approaches in future studies to enhance comparability and generate more generalizable knowledge. Third, some research studies have found that taxi drivers’ mental health is not an independent variable and do not provide sufficient data on possible causes. Additionally, in many of the studied countries, most taxi drivers were migrants, so the results may be influenced by this condition. Factors such as country of origin, language proficiency, under- or over-qualification, lack of a socio-familial network, or racism come into play. A clear example is the research by Davidson et al., who found that the distribution of psychological distress scores among urban taxi drivers was comparable to the distribution reported in studies focusing on immigrant groups from high-conflict countries (Davidson et al. 2018). Finally, the review itself has focused on three specific mental health problems—depression, anxiety, and stress—whereas broader concepts of mental health are increasingly understood within a biopsychosocial framework, extending beyond purely diagnostic categories.

Moreover, in terms of methodological quality, there was considerable variability among the studies. Only one study (9.1%) satisfied more than 50% of the quality criteria, indicating that most of the included studies present significant methodological limitations. Key issues identified in the lower-quality studies included a lack of blinding of assessors, insufficient justification of sample sizes, failure to control for confounding variables, and inconsistencies in the application of inclusion and exclusion criteria. These methodological shortcomings may introduce biases and affect the validity of the results, emphasizing the importance of interpreting the findings with caution.

Future studies on this topic should address key methodological limitations identified in the reviewed studies. These include the lack of sample size calculations, the absence of clear and standardized inclusion and exclusion criteria, and insufficient control for confounding variables. For instance, variables such as age, working hours, years of employment, and experience with night shifts should be systematically recorded and adjusted for in analyses to minimize bias and enhance the reliability of findings. Incorporating these elements would improve study rigor and facilitate more robust comparisons across different contexts.

### Implications for further research

The analysis conducted in this study has identified a number of factors that influence the mental health of taxi drivers, revealing specific challenges and areas for improvement. These recommendations are derived directly from the findings and aim to bridge research and practice. On a scientific level, more research is needed to assess the health of taxi drivers in underrepresented regions and to expand the concept of mental health beyond depression, stress, and anxiety, providing a more comprehensive understanding of their health and associated factors. Longitudinal and prospective studies are necessary to confirm observed associations and establish causal relationships. At the policy level, addressing structural issues such as long working hours and poor working conditions is essential. This could include implementing regulations to limit working hours and promote safer working environments, as well as establishing regular medical check-up programs that include mental health assessments to enable early detection and intervention. At the individual level, our findings highlight the urgency of adopting measures to help taxi drivers manage stress and improve sleep habits. Examples include workshops on stress management, relaxation exercises, sleep hygiene education, and the creation of peer support networks, especially for immigrant drivers who often face additional cultural and social barriers. These proposals should be designed with the social, cultural, and economic realities of taxi drivers in mind, ensuring they are viable even in low-resource settings. Investing in the mental health of this occupational group represents a significant opportunity to reduce suffering, improve health outcomes, enhance quality of life, and positively impact road safety and overall community well-being (World Health Organization 2022a).

## Conclusion

This study aimed to enhance the understanding of mental health of taxi drivers worldwide. Our results showed that depression, anxiety, stress, and psychological distress are significantly higher among taxi drivers compared to the general population. Consequently, this occupational group may be more vulnerable to mental health problems. Investing in the mental health of taxi drivers is not only essential for their individual well-being but also for public health overall. Mentally healthy taxi drivers can provide better service to their passengers and contribute to a safer and healthier society.

## Electronic supplementary material

Below is the link to the electronic supplementary material.

Supplementary Material 1
